# Supplemental ^18^F-FDG-PET/CT for Detection of Malignant Transformation of IPMN—A Model-Based Cost-Effectiveness Analysis

**DOI:** 10.3390/cancers13061365

**Published:** 2021-03-18

**Authors:** Felix Bicu, Johann S. Rink, Matthias F. Froelich, Clemens C. Cyran, Johannes Rübenthaler, Emrullah Birgin, Manuel Röhrich, Fabian Tollens

**Affiliations:** 1 Department of Nuclear Medicine, University Hospital Heidelberg, D-68120 Heidelberg, Germany; felixflorian.bicu@med.uni-heidelberg.de (F.B.); manuel.roehrich@med.uni-heidelberg.de (M.R.); 2 Department of Radiology and Nuclear Medicine, University Medical Center Mannheim, Theodor-Kutzer-Ufer 1-3, D-68167 Mannheim, Germany; matthias.froelich@medma.uni-heidelberg.de (M.F.F.); fabian.tollens@medma.uni-heidelberg.de (F.T.); 3 Department of Radiology, University Hospital, LMU Munich, Marchioninistr. 15, D-81377 Munich, Germany; clemens.cyran@med.uni-muenchen.de (C.C.C.); johannes.ruebenthaler@med.uni-muenchen.de (J.R.); 4 Department of Surgery, Universitätsmedizin Mannheim, Medical Faculty Mannheim, Heidelberg University, D-68167 Mannheim, Germany; emrullah.birgin@umm.de

**Keywords:** cost-effectiveness, IPMN, Pancreas lesions, PET/CT, malignancy detection

## Abstract

**Simple Summary:**

The incidence of IPMN is increasing, mainly attributed to the expanded application of radiological cross-sectional imaging and improvements in image quality. IPMN are the cause of approximately 10% of all pancreatectomies in the USA. A significant number of surgically treated IPMNs do not show high-grade dysplasia or invasive cancer, raising the question of overtreatment, and the need for better diagnostic accuracy. ^18^F-FDG-PET/CT demonstrated promising diagnostic performance in the detection of malignant transformation of IPMN in comparison to CT and MRI. In this study, the authors analyze whether a supplemental ^18^F-FDG-PET/CT to the current diagnostic pathway of IPMN could be cost-effective. Results suggest that implementation of ^18^F-FDG-PET/CT in a preoperative setting could be beneficial from a health care system perspective. It also encourages the research community to investigate if ^18^F-FDG-PET/CT could be a useful addition in other diagnostic settings within IPMN management.

**Abstract:**

Accurate detection of malignant transformation and risk-stratification of intraductal papillary mucinous neoplasms (IPMN) has remained a diagnostic challenge. Preliminary findings have indicated a promising role of positron emission tomography combined with computed tomography and ^18^F-fluorodeoxyglucose (^18^F-FDG-PET/CT) in detecting malignant IPMN. Therefore, the aim of this model-based economic evaluation was to analyze whether supplemental FDG-PET/CT could be cost-effective in patients with IPMN. Decision analysis and Markov modeling were applied to simulate patients’ health states across a time frame of 15 years. CT/MRI based imaging was compared to a strategy with supplemental ^18^F-FDG-PET/CT. Cumulative costs in US-$ and outcomes in quality-adjusted life years (QALY) were computed based on input parameters extracted from recent literature. The stability of the model was evaluated by deterministic sensitivity analyses. In the base-case scenario, the CT/MRI-strategy resulted in cumulative discounted costs of USD $106,424 and 8.37 QALYs, while the strategy with supplemental FDG-PET/CT resulted in costs of USD $104,842 and a cumulative effectiveness of 8.48 QALYs and hence was cost-saving. A minimum specificity of FDG-PET/CT of 71.5% was required for the model to yield superior net monetary benefits compared to CT/MRI. This model-based economic evaluation indicates that supplemental ^18^F-FDG-PET/CT could have a favorable economic value in the management of IPMN and could be cost-saving in the chosen setting. Prospective studies with standardized protocols for FDG-PET/CT could help to better determine the value of FDG-PET/CT.

## 1. Introduction

Intraductal papillary mucinous neoplasms (IPMN) are intrapancreatic mucinous cystic lesions with potential of malignant transformation [1]. Initially defined in 1996 by the World Health Organization (WHO) [2], IPMN are increasingly detected incidental findings, mainly attributed to the expanded application of radiological cross-sectional imaging and improvements in image quality [3,4,5]. 

IPMN are categorized in main duct, branch duct, and mixed type IPMN, each of which is associated with a different risk of malignant change and potential to develop pancreatic ductal adenocarcinoma (PDAC), associated with 5-year survival rates as low as 6% [6,7]. The three current international guidelines for the management of cystic tumors of the pancreas define slightly differing pathways for therapeutic management and surveillance options with regard to IPMN [1,8,9]. For the surveillance of asymptomatic IPMN without worrisome features, different imaging modalities and follow-up intervals have been proposed with contrast-enhanced magnetic resonance imaging (MRI) including magnetic resonance cholangiopancreatography (MRCP) as the modality of choice [1]. Patients diagnosed with IPMN carry a higher overall risk for developing PDAC and the risk of recurrence of IPMN following resection is significant [10]. Therefore, systematic clinical follow-up following surgery is warranted and recommended by the guidelines [1,8,9]. Contrast-enhanced MRI with MRCP provides high diagnostic accuracy for the differentiation of cystic lesions of the pancreas particularly for the depiction of continuity to the pancreatic duct. Yet guidelines also recommend computed tomography (CT) imaging as an alternative. 

At present, the diagnostic accuracy of available procedures for the early detection of high-grade dysplasia (HGD) and invasive cancer in IPMN are not fully satisfactory, with sensitivity and specificity rates reported in the range of 61 to 81% and 52 to 76%, respectively [5,11,12,13,14,15]. 

Positron emission tomography combined with computed tomography (PET/CT) and ^18^F-fluorodeoxyglucose (^18^F-FDG) has gained widespread application for the evaluation of tissue metabolism and identification of malignant tumors by increased glucose metabolic activity [16,17]. The potential of ^18^F-FDG-PET/CT in detecting malignancy in IPMN is promising and has been indicated in recent meta-analyses, with sensitivity and specificity rates of 80 to 95% and 60 to 95%, respectively [11,13,14,15,17], in spite of a small number of studies that could not prove a benefit of FDG-PET/CT in IPMN [18,19]. In particular, technical limitations, such as definition of standardized uptake value cut-offs need to be resolved, and the precise role of additional PET/CT imaging needs to be further delineated as to the patient subgroups and diagnostic contexts that could potentially benefit. Since IPMN account for approximately 10% of pancreatectomies in the United States [5] and pancreatectomy is associated with relevant morbidity and mortality, as well as relevant costs, the impact on patient health, quality of life, as well as healthcare costs is significant [5,20,21].

Cost-effectiveness analyses have gained recognition for healthcare decision makers as they allow for evaluation of both costs and outcomes of innovative medical procedures and facilitate resource allocation decisions [22,23]. The downstream economic value of newly introduced diagnostic procedures might be underestimated in the light of significant short-term costs. This may also be true for the application of supplemental ^18^F-FDG-PET/CT in this patient collective. Therefore, the aim of this economic evaluation was to assess whether supplemental ^18^F-FDG-PET/CT testing for detection of malignant transformation of IPMN could be cost-effective. 

## 2. Materials and Methods

### 2.1. Economic Modeling

#### 2.1.1. Decision Model

**Figure 1:**
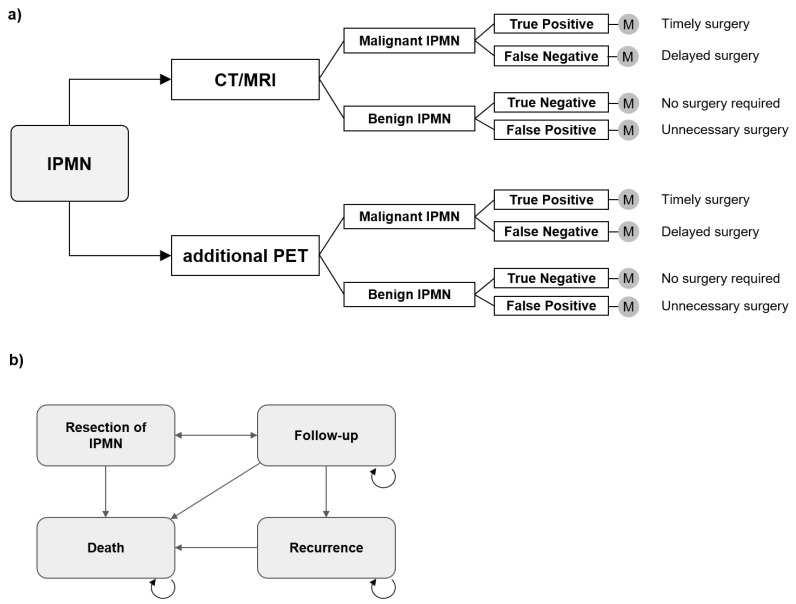
Model structure: (**a**) Decision model for the diagnostic strategies CT/MRI and additional ^18^F-FDG-PET/CT. (**b**) Simplified Markov model.2.2 Input parameters.

Patients with IPMN under evaluation for possible signs of malignancy were subject of analysis. In this context, malignancy is defined as invasive carcinoma or HGD according to European evidence-based guidelines on pancreatic cystic neoplasms and the broadly used definition of many authors [1,9]. Characterization of IPMN and prediction of malignancy were achieved either by CT/MRI or by application of an additional ^18^F-FDG-PET/CT scan, as compared in a recent meta-analysis [15]. The two diagnostic strategies, CT/MRI vs. CT/MRI, and additional ^18^F-FDG-PET/CT, were analyzed in terms of cost-effectiveness (Figure 1a). The corresponding outcomes of the decision model (true positive, false negative, true negative, false positive) were assessed for each diagnostic strategy. In case of positive findings, i.e., signs of malignancy that qualify for resection, pancreatic surgery was conducted, as opposed to negative findings, which resulted in continuing surveillance according to international consensus guidelines. False negative findings resulted in delayed diagnosis, whereas false positive findings resulted in resection of IPMN associated with corresponding costs and impairments in quality of life. 

#### 2.1.2. Markov Model Structure

A Markov model was developed to simulate patients’ health states and associated health care costs using decision analysis and economic modeling software (TreeAge Healthcare Pro. Version 20.1.1, Williamstown, MA, USA). Markov models have proven to be the leading method in evaluating the cost-effectiveness of different diagnostic and therapeutic strategies in health care and consist of a network of health states that are realized with a predefined probability. The model structure is outlined in Figure 1. A cycle length of one year with a time span of 15 years was chosen in order to reflect both short- and long-term outcomes. Follow-up, resection of IPMN and recurrence in case of malignant IPMN were included as health states. Age-adjusted mortality rates were considered, as well as surgery-related mortality and deaths due to recurrent disease. A United States (U.S.) healthcare perspective was taken and the corresponding short- and long-term costs and outcomes were determined in US-$ and quality-adjusted life years (QALY) over the model runtime of 15 years. 

### 2.2. Input Parameters

Input parameters for the study were extracted from literature as explained in Table 1. 

Based on published literature on the diagnostic performance of ^18^F-FDG-PET/CT, the average age at the time of the diagnostic work-up was set to 64.3 years [24,25]. The pre-test probability of malignant IPMN was estimated at 52% [26,27]. Institutional Review Board Statements were available for all studies included in the analysis. Informed consent was not applicable for this study since external clinical data was analyzed.

#### 2.2.1. Diagnostic Efficacy Parameters

The diagnostic performance of conventional imaging (CT/MRI) vs. ^18^F-FDG-PET/CT in detecting malignant IPMN was determined in a recent meta-analysis [15]. A sensitivity and specificity of 80.9 and 76.2% were reported for CT/MRI and of 96.8 and 91.1% for ^18^F-FDG-PET/CT, respectively. 

#### 2.2.2. Utilities and Costs

The quality of life of patients with IPMN was set to 1.0 due to the asymptomatic nature of IPMN which has been observed in the vast majority of patients. Changes in quality of life due to therapeutic measures were considered in the Markov model. Based on the publication of Ljungman et al., the quality of life of patients undergoing pancreatic surgery, long-term follow-up and recurrence of malignant IPMN were adapted and set to 0.818, 0.896, and 0.65, respectively [28,29,30]. The quality of life compared favorably with the QOL reported by other authors like Billings et al. and Epelboym et al. [31,32]. To determine the overall costs of all included procedures from a U.S. healthcare system perspective, costs of diagnostic procedures based on Medicare current procedural terminology (CPT) codes were included. Costs for pancreatic surgery and for management of recurrence were extracted from recent literature [33,34,35,36,37]. 

#### 2.2.3. Transition Probabilities

Age-adjusted risk of death as determined in U.S. Life Tables was used to model average background mortality [38]. Risk of malignant transformation of IPMN, risk of death due to malignant IPMN, recurrence rates and mortality due to recurrences, as well as perioperative mortality in pancreatic surgery were collected from literature [39,40,41]. Risk reduction in recurrence based on early detection was estimated based on expert interviews. 

**Table 1:** Input parameters for the Markov decision model.

Variable	Estimation	Source
Pre-test probability of malignant IPMN	52%	Sugimoto et al., 2017 [26]/Wilson et al., 2017 [27]
Average age at ^18^F-FDG-PET examination	64.3	Hong et al., 2010 [24]/Sperti et al., 2007 [25]
Assumed WTP	$100,000	Sanders et al., 2016 [42]
Discount rate	3.00%	Sanders et al., 2016 [42]
**Diagnostic test performances**		
CT/MRI sensitivity (for risk factors predictive of malignancy)	80.9%	Sultana et al., 2015 [15]
CT/MRI specificity (for risk factors predictive of malignancy)	76.2%	Sultana et al., 2015 [15]
^18^F-FDG-PET sensitivity (for risk factors predictive of malignancy)	96.8%	Sultana et al., 2015 [15]
^18^F-FDG-PET specificity (for risk factors predictive of malignancy)	91.1%	Sultana et al., 2015 [15]
**Costs**		
Contrast-enhanced MRI	$492	Medicare CPT code 74183
^18^F-FDG-PET	$1551	Medicare CPT code 78814
Open pancreatoduodenectomy	$28,623	Gerber et al., 2017 [33]
Distal pancreatic resection	$13,900	Rutz et al., 2014 [34]
Proportion of pancreatic head resection vs. distal pancreatic resection	78%/21%	Mimura et al., 2010 [36]
Cost of recurrent disease	$78,630	Tramontano et al. [37]
Mean cost of readmissions	$1930	Kent et al., 2011 [35]
**Utilities**		
QOL of patients with IPMN	1.00	Assumption
QOL of patients receiving IPMN resection	0.818	Adapted from Ljungman et al., 2011 [29]
QOL of patients with recurrence	0.65	Adapted from Müller-Nordhorn et al., 2006 [28]
Long-term QOL of patients after IPMN resection	0.896	Adapted from Ljungman et al., 2011 [29]
Death	0.00	Assumption
**Transition probabilities**		
Risk of death without malignant IPMN	age-adjusted	US Life Tables 2017 [38]
Risk of malignant transformation	2.23%	Choi et al., 2017 [39]
Risk of death due to malignant IPMN	2.7%	Chari et al., 2002 [41]
Risk of death due to recurrent malignant IPMN	28.3%	Chari et al., 2002 [41]
Perioperative mortality in pancreatic surgery	4.6%	Huang et al., 2010 [40]
Probability of recurrence of malignant IPMN	16.7	Chari et al., 2002 [41]
Reduction in risk of recurrence due to early detection by PET	10%	Assumption

### 2.3. Economic Analysis

#### 2.3.1. Cost-Effectiveness Analysis

Cumulative costs and QALYs were modelled across a time frame of 15 years and discounted at an annual discount rate of 3%. A willingness-to-pay (WTP)-threshold of USD $100,000 per QALY gained was assumed [42] based on international recommendations for cost-effectiveness analyses. The WTP reflects the value of a desired healthcare-related outcome that a society is willing to afford given its economic boundaries. All calculations were carried out in the aforementioned decision analysis software. 

#### 2.3.2. Sensitivity Analysis

The uncertainty of an economic modelling approach can be assessed by sensitivity analyses. Variations of the input variables naturally influence the outcomes of the model.

**Figure 2:**
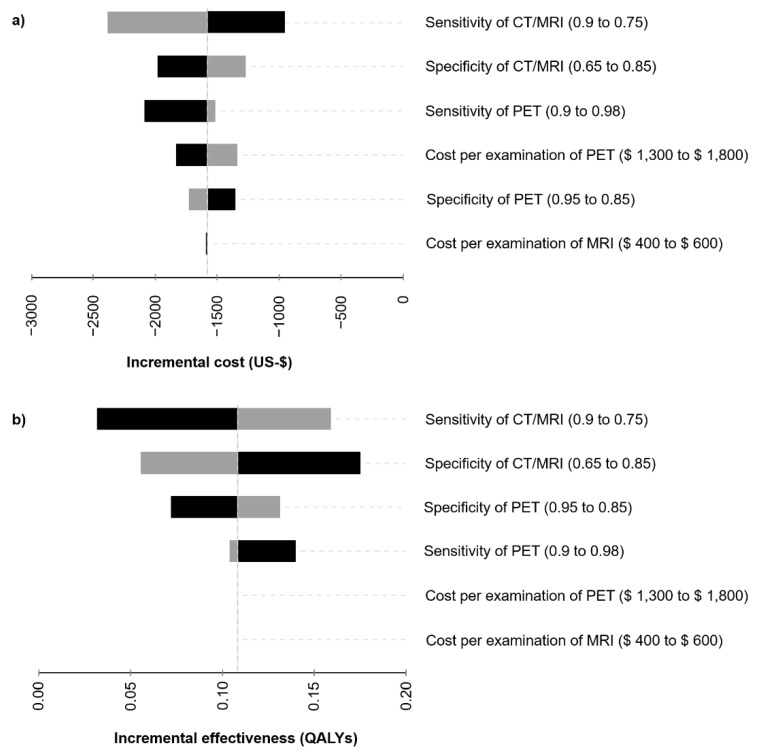
Deterministic sensitivity analysis: (**a**) The influence of various input variables on the incremental cost (US-$) is displayed in a tornado diagram. (**b**) Impact of input variables on the incremental effectiveness expressed by quality-adjusted life years (QALYs) is displayed accordingly. The input variables were varied within reasonable ranges (indicated in the brackets) and the resulting incremental costs and effectiveness of 18F-FDG-PET/CT vs. CT/MRI-strategies were computed.

In a deterministic sensitivity analysis, multiple input variables were varied within a certain range and the impact on the resulting model outputs was studied. Costs of the diagnostic procedures were varied within plausible ranges. Reports on the diagnostic accuracy of ^18^F-FDG-PET/CT and other imaging modalities are heterogeneous. Therefore, these input variables were varied in sensitivity analyses to reflect the range of reported values and to allow for a broader interpretation of results given the uncertainty reported in the literature. The resulting incremental costs and incremental effectiveness based on changes of single variables are visualized in tornado diagrams (Figure 2).

**Figure 3:**
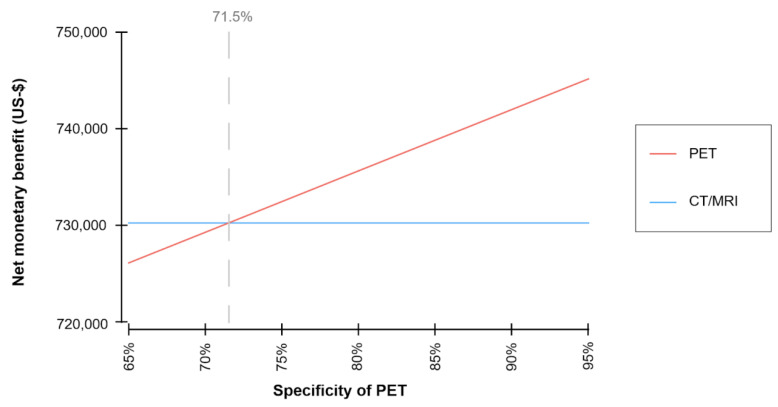
Net monetary benefits of ^18^F-FDG-PET/CT and CT/MRI depending on varying specificity of ^18^F-FDG-PET/CT. For a minimum specificity of ^18^F-FDG-PET/CT of 71.5%, the PET/CT-strategy resulted in superior net monetary benefits (U.S.-$).

Net monetary benefit was simulated for varying specificities of ^18^F-FDG-PET/CT in order to consider uncertainties of the diagnostic performance of ^18^F-FDG-PET/CT in characterizing IPMN, and the corresponding economic value of the strategy (Figure 3). 

### 2.4. Data Availability

The data presented in this study are available from the figures and tables provided. 

## 3. Results

### 3.1. Cost-Effectiveness Analysis

In the base-case scenario, the strategy with an additional ^18^F-FDG-PET/CT resulted in cumulative costs of USD $104,842 and a cumulative effectiveness of 8.48 QALYs, whereas the standard proceedings according to guidelines resulted in costs of USD $106,424 and 8.37 QALYs (Table 2). Assuming a WTP-threshold of USD $100,000 per QALY, net monetary benefit was USD $742,697 for FDG-PET/CT vs. USD $730,272 for CT/MRI. As a result, the FDG-PET/CT-strategy absolutely dominates the standard diagnostic assessment with CT/MRI.

### 3.2. Sensitivity Analysis

To further assess the influence of the input variables on the incremental costs and effects, a deterministic sensitivity analysis was performed (Figure 2). Variations of the costs and performance of the diagnostic procedures consistently resulted in a smaller cost burden and favorable effectiveness of the FDG-PET/CT-strategy compared to CT/MRI. Sensitivity of CT/MRI as a single factor was identified to have the highest impact on incremental costs, whereas both sensitivity and specificity of CT/MRI sensitively affected incremental effectiveness. A specificity of CT/MRI of 65 or 85% would result in an advantage of 0.18 or 0.06 QALYs of the FDG-PET/CT-strategy, respectively. 

Variations of the specificity of ^18^F-FDG-PET/CT have an impact on the net monetary benefit (Figure 3). A minimum specificity of ^18^F-FDG-PET/CT of 71.5% is required for the model to yield superior net monetary benefits for the FDG-PET/CT-strategy.

## 4. Discussion

This model-based cost-effectiveness analysis offers a first economic evaluation of supplemental ^18^F-FDG-PET/CT testing for characterization of IPMN and detection of malignant transformation. The accurate identification of malignancy is crucial in the management of IPMN since pancreatic surgery is associated with significant morbidity and mortality, as well as substantial short-term and long-term cost [20,43]. Apart from CT, MRI and ^18^F-FDG-PET/CT, endoscopic ultrasound assumes an important role as a supplemental diagnostic tool for the detection and characterization of intracystic nodules and carries the advantage of optional fine-needle aspiration (FNA). Cyst fluid analysis provides valuable diagnostic data for further stratification of the sub-entities of cystic pancreatic neoplasms and the assessment of HGD or invasive cancer by biochemical, cellular, and DNA analysis [1,9]. However, FNA is associated with the risk of complications like infection, hemorrhage, and pancreatitis [1,44,45]. Therefore, a non-invasive approach for the early detection of HGD or invasive cancer in IPMN would be beneficial. The results of this model-based economic evaluation provide support to the hypothesis that inclusion of ^18^F-FDG-PET/CT to detect HGD or invasive cancer in IPMN could be cost-saving. The discounted cumulative costs of the PET/CT-strategy offered a small advantage over the CT/MRI-strategy. At the same time, the resulting cumulative QALYs were higher for the PET/CT-strategy, which indicates an absolute dominance of the FDG-PET/CT-strategy in the chosen setting. Lower costs and superior effectiveness of the PET/CT-strategy reflect the superior diagnostic performance of the method expressed by higher sensitivity and specificity. The lower specificity of conventional imaging results in a higher number of false positive findings and consecutive IPMN resections with associated costs and impairments in quality of life. 

The management of IPMN has continuously been in the focus of scientific debate, and international guidelines have repeatedly been revised over the last years [1,44,46]. Improvements in image quality and increasing number of imaging examinations contribute to higher detection rates of IPMN, which consequently resulted in growing numbers of IPMN resections [5,20,47]. At the same time, the limited diagnostic accuracy of current diagnostic modalities for the identification of HGD or invasive cancer in IPMN, that defines further therapeutic management, has been recognized [5,47]. Recent multi-center studies indicated a significant inter-institutional variance of diagnostic performance and available studies investigating the accuracy of current guidelines for the detection of HGD and invasive cancer in IPMN point towards a risk for surgical overtreatment in correlation to the histopathological results of malignancy [12,14]. Improvements in the diagnostic algorithms have the potential to significantly influence IPMN management in the direction of lesion surveillance and an evidence-based and risk-adjusted balance between cancer prevention and surgical overtreatment.

A possible reason for the limited performance may be attributed to the strong reliance of the current consensus guidelines on radiographic criteria on regular follow-up imaging with CT or MRI in surveillance [1,8,9]. Both techniques present a limited level of diagnostic accuracy for the early identification of HGD and invasive cancer in IPMN [11,15]. Pulvirenti et al. demonstrated in their retrospective multi-center study that the number of BD-IPMN resected due to high-risk radiographic features increased following the introduction of the new Fukuoka guidelines 2012, while the rate of high-risk disease among resected IPMN decreased [47]. The review of the current literature together with the significant morbidity and mortality of pancreatic surgery underlines the urgent need for improved diagnostic accuracy of imaging for the sensitive and specific detection of HGD and invasive cancer in IPMN, with particular focus on cost-effectiveness of the employed diagnostic techniques. Promising non-invasive diagnostic modalities arise to close the existing gap in diagnostic certainty regarding risk stratification of IPMN. 

Besides encouraging perspectives on lesion characterization by cyst fluid analysis and advanced DNA sequencing for risk assessment, ^18^F-FDG-PET/CT has been indicated to provide superior diagnostic accuracy in detecting malignant IPMN compared to conventional imaging by CT and MRI [11,13,14,15,19]. However, a recent multi-center study was not able to demonstrate a benefit of ^18^F-FDG-PET/CT with regard to the correct identification of malignant transformation in IPNM [18]. Small sample sizes and lack of standardization in technical procedures and interpretation might contribute to the heterogeneity of reported diagnostic performance of ^18^F-FDG-PET/CT in the assessment of cystic neoplasms of the pancreas.

For the economic evaluation in this study, data were extracted from the meta-analysis by Sultana et al. that included a significant spectrum of studies into the analysis and compared the diagnostic performance of conventional imaging modalities to ^18^F-FDG-PET/CT. The meta-analysis was based on a limited number of studies with overall small sample sizes. However, the reported diagnostic performance was well in line with other recent meta-analyses that consistently deemed ^18^F-FDG-PET/CT the superior modality [11,13,14]. Only recently, Liu et al. concluded in a meta-analysis, that FDG-PET/CT imaging offered the highest sensitivity for malignancy detection, whereas diffusion-weighted magnetic resonance imaging (DWI-MRI) offered the highest specificity [19]. The authors recommended the use of MRI or PET/CT as suitable first-line diagnostic modalities for detection of IPMN. Overall, further research is required to identify subgroups of patients that could potentially benefit most from supplemental ^18^F-FDG-PET/CT. Additional diagnostics might prove particularly useful in cases without high-risk features or in branch duct or mixed type IPMN with suspected worrisome features. 

In order to analyze the impact of uncertainty of the input variables, sensitivity analyses have been conducted. When specificity of ^18^F-FDG-PET/CT was reduced below 71.5%, the strategy no longer provided an advantage in terms of net monetary benefit. These findings indicate that the superior economic value of the ^18^F-FDG-PET/CT-strategy as determined by this model-based analysis depends on its assumptions, i.e., the superior diagnostic accuracy of ^18^F-FDG-PET/CT. This finding is supported by Sharib et al., who were able to demonstrate that current guidelines were not cost-effective for the management of pancreatic cysts and reported a minimum specificity of 67% for guideline-based surveillance to be cost-effective compared to surgery or a watch-and-wait-strategy [20].

Further limitations of this model-based approach deserve closer scrutiny. The results derived from the presented Markov model need to be carefully interpreted in their clinical context, bearing in mind the limited availability of clinical data. A Markov model will never accurately reflect any clinical situation, but represents a simplified model of the examined clinical decision that has to rely on certain assumptions. A United States healthcare system perspective was chosen with all costs estimated in US-$. Due to the limited availability of data, a distinction between subtypes of IPMN, i.e., branch duct, main duct and mixed-type IPMN, could not be introduced into the chosen Markov Model. Therefore, interpretation and applicability of the results with respect to subgroups of patients is limited. 

First data indicate the potential of ^18^F-FDG-PET/MRI for the assessment of IPMN, which must yet be considered a realm of future research [48,49,50]. 

In this study, cost-effectiveness of single time point additional ^18^F-FDG-PET/CT testing for the detection of malignancy of IPMN was evaluated. The role of ^18^F-FDG-PET/CT in screening and surveillance strategies is a matter of future research. So far, data on the repeated use of ^18^F-FDG-PET/CT in surveillance of IPMN are missing and were not the subject of this analysis.

## 5. Conclusions

This cost-effectiveness analysis indicates that supplemental ^18^F-FDG-PET/CT testing has promising economic value and could be cost-saving in the chosen setting, assuming superior diagnostic performance for the detection of HGD and invasive cancer in IPMN compared to conventional imaging as concluded by recent meta-analyses. However, further evaluation of the additional value of ^18^F-FDG-PET/CT for the detection of malignant change of IPMN in larger patient collectives is required. The findings of this study contribute to the understanding of ^18^F-FDG-PET/CT as an attractive candidate for further investigation based on considerations of cost-effectiveness. 

## Figures and Tables

**Table 2:** Results of the base-case cost-effectiveness analysis comparing CT/MRI with additional ^18^F-FDG-PET/CT examination. Cumulative discounted costs and effectiveness for a time frame of 15 years.

Strategy	Cumulative Discounted Costs (US-$)	Incremental Costs (US-$)	Cumulative Discounted Effectiveness (QALYs)	Incremental Effectiveness (QALYs)	Net Monetary Benefit (US-$)
Add. ^18^F-FDG- PET/CT	$104,842	n/a	8.48	n/a	$742,697
CT/MRI	$106,424	$1581	8.37	−0.11	$730,272

## Data Availability

Data is contained within the article.
